# *In Silico* Analysis of Putrefaction Pathways in Bacteria and Its Implication in Colorectal Cancer

**DOI:** 10.3389/fmicb.2017.02166

**Published:** 2017-11-07

**Authors:** Harrisham Kaur, Chandrani Das, Sharmila S. Mande

**Affiliations:** Bio-Sciences R&D Division, TCS Research, Tata Consultancy Services Ltd., Pune, India

**Keywords:** protein fermentation, genome mining, bacterial pathogenicity, gut microbiome, colorectal cancer

## Abstract

Fermentation of undigested proteins in human gastrointestinal tract (gut) by the resident microbiota, a process called bacterial putrefaction, can sometimes disrupt the gut homeostasis. In this process, essential amino acids (e.g., histidine, tryptophan, etc.) that are required by the host may be utilized by the gut microbes. In addition, some of the products of putrefaction, like ammonia, putrescine, cresol, indole, phenol, etc., have been implicated in the disease pathogenesis of colorectal cancer (CRC). We have investigated bacterial putrefaction pathways that are known to be associated with such metabolites. Results of the comprehensive *in silico* analysis of the selected putrefaction pathways across bacterial genomes revealed presence of these pathways in limited bacterial groups. Majority of these bacteria are commonly found in human gut. These include *Bacillus, Clostridium, Enterobacter, Escherichia, Fusobacterium, Salmonella*, etc. Interestingly, while pathogens utilize almost all the analyzed pathways, commensals prefer putrescine and H_2_S production pathways for metabolizing the undigested proteins. Further, comparison of the putrefaction pathways in the gut microbiomes of healthy, carcinoma and adenoma datasets indicate higher abundances of putrefying bacteria in the carcinoma stage of CRC. The insights obtained from the present study indicate utilization of possible microbiome-based therapies to minimize the adverse effects of gut microbiome in enteric diseases.

## Introduction

Putrefaction inside human gastrointestinal tract (gut) pertains to decomposition or fermentation of undigested proteins by the resident microbiota (Windey et al., 2012). The dietary proteins that escape digestion/absorption in small intestine and reach distal (or large) intestine, act as substrates for bacterial fermentation (Yao et al., 2016). Once such undigested proteins are broken down into amino acids in the large intestine, they are usually metabolized by the resident proteolytic bacteria, leading to production of harmful metabolites (Hughes et al., 2000; Windey et al., 2012; Yao et al., 2016). The excess products of this process have also been reported to be mostly detrimental to gut health, unlike those of carbohydrate fermentation which help maintaining gut homeostasis (Conlon and Bird, 2014). Such products include ammonia, amines (like putrescine), cresol, indole, phenol, etc. (Windey et al., 2012; Yao et al., 2016). Earlier studies have proposed roles of these metabolites in the pathogenesis of gastrointestinal diseases (Hughes et al., 2000; Kim et al., 2013; Silva et al., 2015; Yao et al., 2016). Given the clinical importance of putrefaction products, an analysis of putrefaction pathways utilized by various gut bacteria is likely to be helpful for obtaining insights into possible strategies used by the bacterial groups for exerting harmful effects on gut.

The deleterious effects of putrefaction (in gut) have been reported to be associated with colorectal cancer (CRC) (Hughes et al., 2000; Kim et al., 2013). For example, ammonia, one of the products of putrefaction, has been suggested to promote intestinal cell proliferation and also to aid growth of cancer cells over normal cells in CRC (Lin and Visek, 1991). Results from an earlier study have also indicated that colonic cells get damaged due to increased absorption of ammonia when they get exposed to ammonia for prolonged period (Fung et al., 2013). Similarly, two other putrefaction products, namely, phenol and p-cresol have been associated with increase in colonic DNA damage (Toden et al., 2005). The polyamines, like putrescine, spermidine, spermine, and cadaverine, produced through fermentation of amino acids (lysine and arginine) have been suggested to be involved in the tumorigenesis of CRC (Gerner, 2007; Minois et al., 2011; Pegg, 2013; Vargas et al., 2015). Further, another putrefaction product, hydrogen sulfide (H_2_S) has been reported to cause disruption of colonocyte barrier function (Carbonero et al., 2012). Thus, the detrimental effect of these putrefaction products, especially in development of CRC, is now fairly acknowledged.

Colorectal cancer is one of the leading causes of cancer related deaths worldwide (Jemal et al., 2011). Diagnosis at early stage has been suggested to be challenging and critical as the survival rate drastically falls upon invasion and initiation of metastasis (Zackular et al., 2014). The major factors believed to influence the disease etiology of CRC include genetics, diet, and environment (Tuan and Chen, 2016). Recent studies have also indicated dysbiosis in gut microbiome to be associated with CRC (Wang et al., 2012; Wu et al., 2013; Zackular et al., 2014). With increasing number of studies delineating the relation of altered gut microbiome with intestinal diseases like CRC, one of the facets of clinical research is toward unraveling different aspects of the aforementioned association. Thus, understanding the role of putrefaction capabilities of microbiome in the gut of CRC patients is likely to shed light on the deleterious effects of putrefaction products on gut health.

In the present study, a comprehensive analysis of bacterial genomes was performed to predict their capabilities of utilizing selected putrefaction pathways that are known to be associated with harmful metabolites. The presence of putrefaction pathways in various bacteria was predicted based on parameters like homology of constituent enzymes, enzyme specificity and genomic proximity of corresponding genes. Apart from obtaining the overall distribution of putrefaction pathways across different bacterial groups, the results of the current study indicate importance of the putrefaction pathways in bacteria commonly associated with gut environment. In addition, the current observations suggest involvement of some of these pathways in bacterial pathogenicity. Further, the insights obtained from genome mining have been utilized to understand the role of these pathways harbored by the gut microbiome in CRC patients.

## Materials and Methods

### Selection of Putrefaction Pathways

The primary focus of the current study pertains to bacterial putrefaction pathways which lead to production of compounds that have been reported to be detrimental to gut. Several *in vivo*/*in vitro* studies have demonstrated the harmful effects of some of the products of amino acid fermentation (putrefaction) on gut. These include ammonia, H_2_S, amine (like putrescine, spermidine, spermine, and cadaverine), cresol, indole, and phenol (Louis et al., 2014; Neis et al., 2015; Yao et al., 2016). For example, such amines have been implicated in DNA damage and tumorigenesis of gastric and colon cancer (Gerner, 2007; Minois et al., 2011; Pegg and Casero, 2011). In addition, *N*-nitroso compounds (NOCs), which can be formed through nitrosation of amines, have been suggested to exhibit carcinogenic effects (Hughes et al., 2000). The metabolic pathways for NOCs have widely been studied in the context of nitrate assimilation by gut bacteria. Thus, the bacterial putrefaction pathways leading to production of the above mentioned nine compounds have been considered in the present study. It may be noted that ammonia is released during the catabolism of any amino acid in the initial deamination step. Consequently, bacteria capable of fermenting any amino acid would release ammonia during the process. This ammonia can enter the urea cycle and subsequently be excreted under normal condition. However, certain conditions (like high protein and low fiber diet) may lead to production of excess ammonia that can damage colonic cells (Fung et al., 2013). Therefore, mere presence of any ammonia releasing pathways, in most cases, is probably not predictive of the harmful effects of the corresponding bacteria. Thus, in the current study, a set of three ammonia releasing pathways [histidine → glutamate, histidine → tetrahydrofolate (THF) and glutamate → acetate + pyruvate], previously reported to be functional in one of the pathogenic gut bacteria (*Fusobacterium*), has been selected for further analysis. Although phenylacetic acid and branched chain fatty acid (BCFA) have been suggested to be efficient indicators of amino acid fermentation in the colon (Louis et al., 2014; Yao et al., 2016), we have not considered them since to the best of our knowledge, these compounds have not been reported to have any harmful effect on the gut. The available literature data on the pathways corresponding to the selected above mentioned nine compounds was further surveyed and utilized for prediction of these pathways across bacterial genomes.

### Collating Information on Experimentally Identified Putrefaction Pathways Associated with Release of Harmful Compounds

Prediction of a pathway in an organism can be performed by mapping the homologs of its constituent enzymes. However, some pathways may contain one or more enzymes that have generic function (like dehydrogenase) which participate in multiple functional pathways. Thus, identification of a particular pathway based on only presence of homologs may lead to false predictions. Therefore, in the current study, in addition to enzyme homology, the genomic proximity of the constituent genes has been utilized for prediction of a pathway. However, it may be noted that the genomic proximity (of the constituent genes) may not be true for all pathways. Therefore, in the current study, the putrefaction pathways leading to production of the selected nine metabolites (as discussed in the previous section) were further filtered to shortlist the following three categories of pathways-

I Pathways where proximity of the constituent genes have been reported by previous experimental studies.
II Pathways where proximity have been reported for a subset of the constituent genes by previous experimental studies.
III A third category of pathways has been considered, where the constituent genes may not be located in close proximity, but are catalyzed by enzymes specific to the corresponding pathways.

For each of the harmful metabolites, the experimentally validated putrefaction pathways (Supplementary Table S1) that belong to the above three categories are described below.

#### (a) Ammonia

**FIGURE 1:**
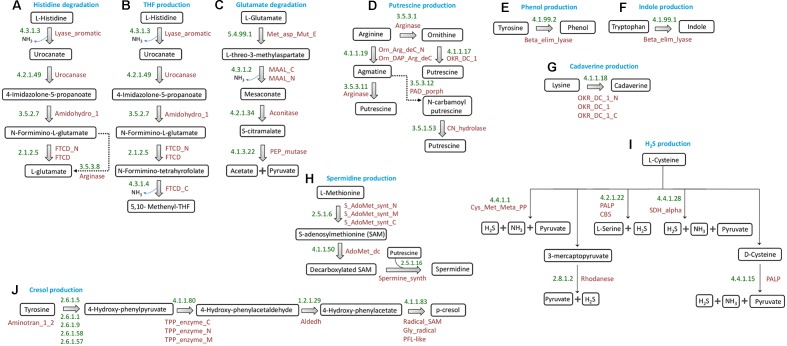
Schematic representation of putrefaction pathways. Pathways for conversions of **(A)** histidine to glutamate, **(B)** histidine to tetrahydrofolate, **(C)** glutamate to acetate and pyruvate, **(D)** arginine to putrescine, **(E)** tyrosine to phenol, **(F)** tryptophan to indole, **(G)** lysine to cadaverine, **(H)** Methionine to spermidine/spermine, **(I)** cysteine to H_2_S, and **(J)** tyrosine to cresol. The EC numbers and Pfam domain(s) corresponding to the enzymes involved in each reaction have been mentioned.

Among the three ammonia releasing pathways initially selected for analysis (as discussed in section “Selection of Putrefaction Pathways”), the enzymes participating in two pathways (histidine → glutamate and histidine → THF) (**Figures 1A,B**, respectively) have been reported to be encoded by gene clusters in *Fusobacterium nucleatum* ATCC 25586 (Kastenmüller et al., 2009). The identification of histidine utilization locus in *Pseudomonas fluorescens* SBW25 by earlier studies further confirms the occurrence of gene cluster encoding the enzymes of histidine degradation pathway in bacteria (Zhang and Rainey, 2007). These two pathways have been referred to as ‘histidine degradation’ and ‘THF production’ pathways throughout the manuscript. Further, for the glutamate fermentation pathway (glutamate → acetate + pyruvate) (**Figure 1C**), the third ammonia releasing pathway considered here, a subset of the constituent genes has been reported to occur in genomic context in *Fusobacterium varium* (Ramezani et al., 2011). The genes encoding two (EC: 5.4.99.1 and EC: 4.3.1.2) out of the four enzymes of the pathway are located in consecutive positions in the *F. varium* genome (Ramezani et al., 2011). The remaining two genes (encoding the enzymes EC: 4.2.1.34 and EC: 4.1.3.22) were found in distant genomic locations. This pathway has been referred to as ‘glutamate degradation’ in the subsequent sections.

#### (b) Putrescine

Out of the three sub-pathways for production of putrescine from arginine (**Figure 1D**), the one through ‘ornithine’ has been reported to be functional in organisms like *Lactobacillus* sp. 30a, *Oenococcus oeni* and *Lactobacillus brevis* IOEB 9906 (Coton et al., 2010; Romano et al., 2014). In these organisms, the corresponding enzymes are encoded by genes which are located in genomic proximity. In the remaining two sub-pathways for putrescine production, arginine is first converted to agmatine by the enzyme ‘arginine decarboxylase’ (ADC; EC 4.1.1.19). This agmatine can then follow one of the two routes leading to production of putrescine. In *Escherichia coli*, the enzyme ‘agmatinase’ (EC: 3.5.3.11), encoded by a gene operonic to ADC, converts agmatine to putrescine and urea (Satishchandran and Boyle, 1986). The other sub-pathway has been reported to be functional in organisms belonging to the genus *Pseudomonas*, where the two corresponding enzymes namely, agmatine deiminase (EC: 3.5.3.12) and carbamoylputrescine hydrolase (EC: 3.5.1.53) are encoded by the operon aguBA (Nakada et al., 2001). The former converts agmatine to *N*-carbamoyl putrescine, which further gets converted to putrescine by the later enzyme. The genes encoding these two enzymes are operonic and are located in distant position from the gene encoding ADC.

#### (c) Spermidine and Spermine

Putrescine can lead to production of another polyamine spermidine with the help of the enzyme spermidine synthase (SpeE, EC: 2.5.1.16) (**Figure 1H**). In addition, a product of methionine catabolism, decarboxylated *S*-adenosyl methionine (decarboxylated SAM), acts as a cofactor for spermidine synthesis (Xie et al., 1989). The pathway for production of this cofactor is methionine →*S*-adenosyl methionine → decarboxylated SAM. While the former reaction is catalyzed by the enzyme MetK (EC: 2.5.1.6), the latter reaction depends on SpeD (EC: 4.1.1.50). SpeD and SpeE have been reported to be encoded by two operonic genes (Xie et al., 1989). The enzyme SpeE can also convert spermidine to spermine (Shah and Swiatlo, 2008).

#### (d) Cresol

The pathway for production of cresol (**Figure 1J**) has been reported to be functional in *Clostridium difficile*, where tyrosine is broken down into the intermediate para-hydroxyphenylacetate (p-HPA) by genes of hpdBCA operon (Dawson et al., 2011). The three genes of this operon, *hpdA, hpdB*, and *hpdC* encode for activating, large and small subunits, respectively, of the corresponding enzyme 4-hydroxyphenylacaetate decarboxylase (EC: 4.1.1.83).

#### (e) Indole, Phenol, Cadaverine, and H_2_S

The pathways for production of indole (from tryptophan), phenol (from tyrosine), and cadaverine (from lysine), each involves single step reaction catalyzed by specific enzymes (Kumagai et al., 1970; Snell, 1975; Park et al., 2017). Further, H_2_S has been reported to be produced from cysteine by any of the five specific enzymes (Carbonero et al., 2012). The reactions corresponding to production of indole, phenol, cadaverine and H_2_S have been depicted in **Figure 1**.

Thus, based on the above information, 10 putrefaction pathways corresponding to the production of nine metabolites were shortlisted for further analysis.

### Identification of Bacterial Putrefaction Pathways Associated with Release of Harmful Compounds

The methodology used for prediction of the selected 10 putrefaction pathways across bacterial genomes has been described below.

#### Pathways Having Constituent Genes in Proximity

Prediction of 6 out of 10 selected putrefaction pathways (producing ammonia/putrescine/cresol/spermidine/spermine) with enzymes encoded by gene clusters included two major steps, namely, (i) identification of homologs of the gene components of putrefaction pathways (ii) demarcation of gene clusters based on genomic distances. Although available algorithms like antiSMASH and ClusterFinder utilize gene context information for prediction of bacterial pathways/function, they primarily focus on identification of cluster of genes involved in biosynthesis of specific group of metabolites (such as secondary metabolites) (Medema et al., 2011; Cimermancic et al., 2014). For prediction of the specific pathway, while antiSMASH uses genomic proximity (similar to our approach), ClusterFinder employs a supervised learning method based on substantial data on experimentally identified gene clusters. Thus, these methods are unsuitable for the prediction of putrefaction pathways under study. Further, unlike the above mentioned methods, although C-Hunter algorithm can be applied for prediction of any pathway, it depends on the functional annotation of genes (like GO annotation) corresponding to the pathway of interest (Yi et al., 2007). In contrast, the present study does not depend on any prior information on functional characterization of the gene components while predicting the putrefaction pathways from bacterial genomes. Our approach rather employs a more unsupervised method, primarily based on similarities of protein domains of the constituent genes and their genomic proximity.

The first step of the methodology adopted in the present study corresponds to identification of homologs by utilizing information pertaining to the domains of the constituent proteins of the selected putrefaction pathways. This approach was used for capturing distant homologs of gene products having low global sequence similarity. For this purpose, the protein sequences corresponding to the enzymes of experimentally detected putrefaction pathways (from the organisms listed in Supplementary Table S1) were queried against ‘Pfam database’ (Finn et al., 2014) and the corresponding Pfam domains were retrieved (Supplementary Table S2). The Hidden Markov Model (HMM) profiles of these ‘seed’ domains were stored in a database. The protein sequences corresponding to all completely sequenced 2738 bacterial genomes^1^) were then queried against this database using the module ‘hmmscan’ provided in the package ‘HMMER’ (version 3.1) (Eddy, 1998) with an e-value threshold of 1e-06. The hits (protein sequences) obtained through ‘hmmscan’ were further analyzed for genomic proximity of the gene components. The genomic proximity was assessed for all the genes while predicting the pathways of ‘category I’ (histidine degradation, THF production, ornithine, and agmatinase sub-pathways of putrescine production). On the other hand, the ‘category II’ pathways (glutamate degradation, agmatine deiminase sub-pathway of putrescine production, spermidine/spermine production) were predicted based on presence of a subset of genes in proximity along with presence of homologs of the remaining distantly located genes. For the remaining fifth pathway under ‘category II,’ i.e., cresol production pathway, the domain information corresponding to one of the enzyme subunits (small subunit) was found to be uncharacterized in Pfam database. Thus, to handle such gaps in the existing knowledge-base, the list of organisms containing the enzyme complex involved in cresol production was retrieved from ‘Uniprot’ database^2^.

#### Pathways Containing Single Reaction Catalyzed by Specific Enzyme

The genomic context based information was not required for prediction of the pathways of ‘category III’ (production of phenol, indole, cadaverine, and H_2_S) since each of these pathways involve single step reaction mostly catalyzed by specific enzymes (**Figures 1E–G,I**). Thus, the organisms containing the corresponding enzymes were retrieved from ‘Uniprot’ database^3^.

### Evaluation of Putrefaction Capability of Bacteria

One of the primary objectives of the current study pertains to analysis of gut microbiome for delineating any probable association of the aforementioned microbial (putrefaction) pathways with CRC. Since, the currently available technologies do not allow the identification of all bacteria in a given metagenome at strain level, it was necessary to obtain measures of putrefaction capabilities for bacterial groups at higher taxonomic levels. Thus, based on the proportion of constituent putrefying strains and a confidence value of the corresponding group, a score was evaluated at the levels of phylum and genus. The confidence value was incorporated to assign a higher weightage to the bacterial group having relatively higher representative organisms in the database. For evaluating the confidence score, the number of strains under all bacterial groups was first noted. Based on these counts a percentile value was computed for each of the groups. Subsequently, these percentile values were used to assign ranks (between one and five) to the groups. The final putrefaction score (Pfacs), for a particular putrefaction pathway ‘i’ corresponding to a bacterial group ‘j,’ was calculated using the following equation-

$${\text{Pfacs}}_{ij}=S*\alpha$$

where, *S* represents the proportion of putrefying organisms of the particular bacterial group and α denotes the confidence value of the corresponding bacterial group.

Thus, the values of the computed ‘Pfacs’ scores ranged between ‘zero’ and ‘five.’ For a particular putrefaction pathway, a bacterial taxon having a higher ‘Pfacs’ would indicate a greater probability of presence of that pathway in the corresponding strains as opposed to a taxon with a lower ‘Pfacs.’

### Identification of Putrefaction Pathways (Associated with Release of Harmful Compounds) in Gut Pathogens and Commensals

In order to investigate the association between bacterial pathogenicity and putrefaction capability, comprehensive list of known gut commensals and pathogens was collated from ‘Integrated Microbial Genomes and Microbiome’ (IMG/M) (Chen et al., 2017) and literature (Supplementary Table S3). The gut strains affiliated as pathogens and commensals were then mapped to the catalog of putrefactors identified in the current study and the respective distributions of putrefaction pathways were obtained.

### Comparison of Putrefaction Pathways (Associated with Release of Harmful Compounds) in Gut Microbiome of Colorectal Cancer and Healthy Individuals

In order to compare the putrefaction capabilities of the microbes residing in the gut of CRC patients with that of healthy individuals, the gut microbiome data (16S rRNA sequences) provided in five published studies on CRC was analyzed (Kostic et al., 2012; Wang et al., 2012; Zackular et al., 2014; Burns et al., 2015; Nakatsu et al., 2015). Details on the analyzed datasets are provided in Supplementary Table S4. The ‘sra’ files obtained from the datasets under study were extracted using SRA toolkit 2.3.4 (Leinonen et al., 2011) and the retrieved fastq sequences were subjected to quality filtration. Prinseq-lite (Schmieder and Edwards, 2011) was utilized to obtain the sequences (in ‘FASTA’ format) having an average phred quality score of more than or equal to 25 for further downstream analyses. Subsequently, Naive Bayesian classifier of the Ribosomal Database Project (RDP classifier 2.10) (Wang et al., 2007) was utilized for taxonomic assignment of the sequences at a bootstrap confidence threshold of 80% for phylum, class, order, family and genus levels. In-house scripts were used to generate abundance of each taxon for healthy, adenoma and carcinoma samples. The obtained taxonomic abundances were normalized to estimate the relative abundances of taxa in each sample.

For identification of differentially abundant taxa in healthy, adenoma and carcinoma samples, multivariate analysis was performed on the normalized abundance for each taxon. Studies containing two distinct classes or groups (healthy and carcinoma) were subjected to Welch *t*-test (Welch, 1938). Kruskal–Wallis test (Kruskal and Wallis, 1952) was performed to identify differential bacterial groups in studies comprising three sets of cohorts (healthy, adenoma, and carcinoma). The genera that appeared as significantly different (in terms of abundance with a *p*-value ≤ 0.05) were identified as differentially abundant genera in the corresponding cohort. Subsequently, the differentially abundant genera of CRC cohort (corresponding to carcinoma as well as adenoma stages) were compared with those of healthy population, in terms of – (i) abundances of putrefying bacteria and (ii) distribution of putrefaction pathways.

### Correlation between Hypoxic Tumor Micro-environment and Putrefying Bacteria

The advanced stage of CRC has been associated with hypoxic (oxygen deficit) tumor micro-environment and subsequent growth of anaerobic pathogenic bacteria (Cummins and Tangney, 2013; Scanlon and Glazer, 2015). In order to gain insights into the proportion of putrefactors that may promote tumor aggravation by contributing to the virulence of the anaerobic pathogens, the differentially abundant taxa in healthy, adenoma and carcinoma were further analyzed from the perspective of their oxygen requirement. For each of the differentially abundant genera in healthy, adenoma and carcinoma cohorts of different studies, data on their oxygen requirement were collected from literature. The differential genera were then classified into five categories, namely, aerobe, anaerobe, microaerophile, facultative anaerobe, and obligate anaerobe. The strains belonging to genera like *Streptococcus* have been reported to be either obligate or facultative anaerobes. Thus, for such cases, a generic classification of ‘anaerobe’ was assigned. Further, the proportion of putrefactors in each of the above mentioned categories were noted.

## Results

### Bacterial Putrefaction Pathways Associated with Release of Harmful Compounds

**FIGURE 2:**
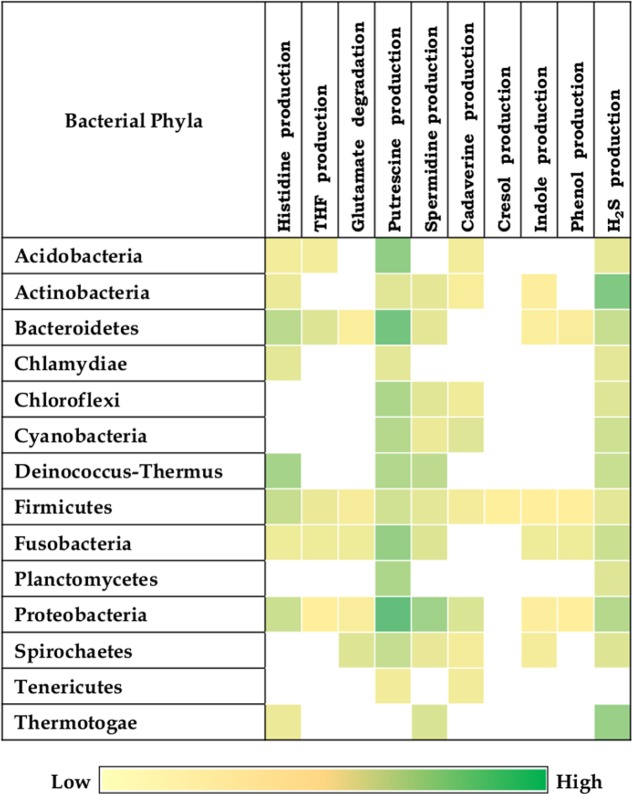
Putrefaction pathways across bacterial phyla. Distribution of 10 putrefaction pathways across phyla comprising of completely sequenced bacterial genomes. The pathway ‘Spermidine production’ also represents the pathway for spermine production. ‘Pfacs’ (Putrefaction score) corresponding to the putrefaction pathways in these phyla is highlighted in each cell. ‘Pfacs’ indicates the relative putrefaction capabilities of any phylum, evaluated based on the number of constituting putrefying strains and the database size of the respective phylum.

The results indicate presence of putrefaction pathways in 1368 (50%) bacteria out of the analyzed 2,738 completely sequenced genomes (Supplementary Data Sheet S1). One or more of the selected putrefaction pathways were observed in bacteria belonging to 14 phyla (**Figure 2**). The distributions of the selected 10 putrefaction pathways which lead to release of nine compounds (ammonia, putrescine, spermidine, spermine, cresol, indole, phenol, cadaverine, and H_2_S) indicate presence of all the 10 pathways in Firmicutes. Considering the cumulative contribution of putrefying strains belonging to a phylum, while Proteobacteria was predicted to have nine pathways (except tyrosine → cresol), Fusobacteria and Bacteroidetes, were predicted to contain eight pathways (except tyrosine → cresol and lysine → cadaverine). Further, it is noteworthy that, although the strains of Firmicutes cumulatively represented all the 10 pathways, the corresponding ‘Pfacs’ were relatively lower (≤1.4). This suggests that, the putrefaction capability of the phylum Firmicutes is limited to selected members. In contrast, the phyla Acidobacteria, Actinobacteria, Bacteroidetes, Fusobacteria, and Proteobacteria were observed to have comparatively higher ‘Pfacs’ of at least half of the maximum value (2.5) in one or more of the pathways. Interestingly, bacteria belonging to five (Firmicutes, Actinobacteria, Bacteroidetes, Fusobacteria, and Proteobacteria) out of six above mentioned phyla have been reported to be common inhabitants of the human gut (Rajilić-Stojanović et al., 2007; Zhang et al., 2015). Organisms belonging to the sixth phylum (Acidobacteria) have mostly been found in soil (Jones et al., 2009; Zhang et al., 2014). However, earlier studies have reported presence of some of its strains in human gut (Stearns et al., 2011). This suggests the possible role of bacterial putrefaction in the gut, where availability of undigested proteins can trigger activation of certain putrefaction pathways in the above mentioned bacterial groups.

### Gut Bacteria and Putrefaction Pathways Associated with Release of Harmful Compounds

**FIGURE 3:**
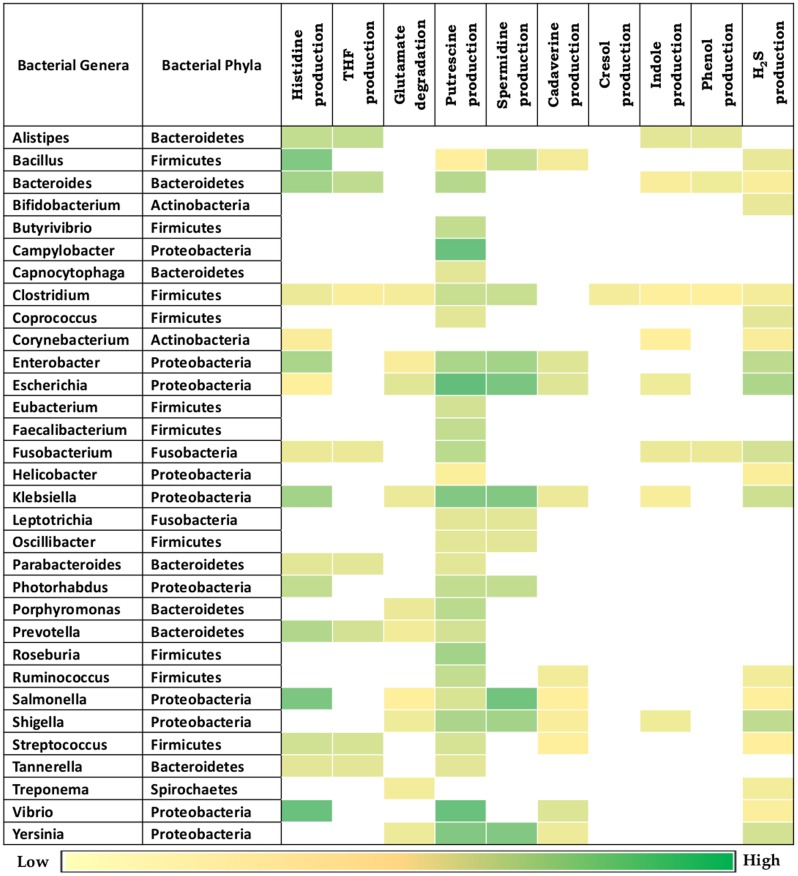
Putrefaction pathways in bacterial genera found in gut. Distribution of ten putrefaction pathways across genera comprising of completely sequenced bacterial genomes found in human gut. The pathway ‘Spermidine production’ also represents the pathway for spermine production. ‘Pfacs’ (Putrefaction score) corresponding to the putrefaction pathways in these genera is highlighted in each cell. ‘Pfacs’ indicates the relative putrefaction capabilities of any genus, evaluated based on the number of constituting putrefying strains and the database size of the respective genus.

Given the observed association of the selected bacterial putrefaction pathways and gut environment, further analysis was performed on the bacterial genera previously known to be associated with gut (**Figure 3**). The results indicate that, the strains belonging to some of the genera contain diverse combinations of analyzed putrefaction pathways, similar to the trend observed in phyla level analysis. This suggests that these pathways may not have been evolutionary retained, but rather have been acquired by the respective organisms for adaptation to the environment they survive in. Overall, the putrescine production pathway (arginine → putrescine) was observed to have highest occurrence (87.5%) among all other pathways (**Figure 3**). Putrescine like polyamines have been reported to be involved in functions like growth, cell wall synthesis, cell signaling, biofilm formation in bacteria (Wortham et al., 2007). Thus, the present results indicate the probable role of this pathway in growth and survival of putrescine producing bacteria. The next highest occurring putrefaction pathways (following the putrescine production pathway) corresponded to histidine degradation (histidine → glutamate) and H_2_S production (cysteine → H_2_S) with 50% and 56% occurrence, respectively. Among others, the occurrence of five pathways for production of THF (from histidine), glutamate (from histidine), indole (from tryptophan), spermidine/spermine (from methionine) and cadaverine (from lysine) were observed to vary between 25% and 35%. The remaining two pathways corresponding to phenol (from tyrosine) and cresol (from tyrosine) production were predicted to be present in limited number of genera (12.5% and 3%, respectively). The genera observed to have higher ‘Pfacs’ (≥2.5) for at least one pathway include *Bacillus, Bacteroides, Enterobacter, Escherichia, Klebsiella, Prevotella, Salmonella, Shigella, Vibrio*, and *Yersinia*. In addition, the group of representative strains of Firmicutes phylum, having as much as nine pathways including exclusive presence of cresol production pathway (as mentioned in previous paragraph) was observed to belong to the genus *Clostridium*. Earlier studies have also suggested the ability of production of cresol through tyrosine fermentation to be unique to *Clostridium* genus (Selmer and Andrei, 2001). Further, the genera *Escherichia* and *Klebsiella* were predicted to harbor seven of the putrefaction pathways. This was followed by *Fusobacterium, Enterobacter, Salmonella*, and *Shigella*, which were observed to possess six out of the 10 putrefaction pathways. Thus, the current results indicate that the above mentioned genera have the capability to ferment various amino acids (like histidine, glutamate, arginine, lysine, cysteine, tyrosine, and tryptophan), acquired from undigested proteins that escape energy metabolism and consequently are likely to affect the gut functioning. In addition, since some of the strains of the above mentioned genera have been reported to exert pathogenicity in gut, further analysis of putrefaction pathways in pathogenic and commensal bacteria were carried out.

**FIGURE 4:**
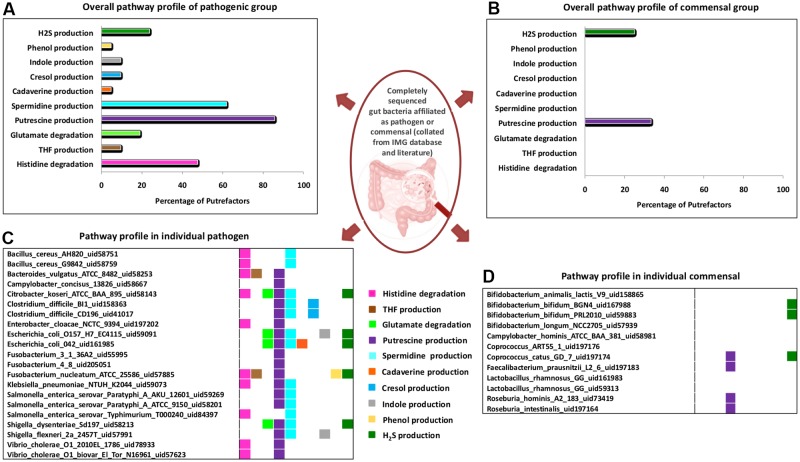
Distribution of putrefaction pathways in pathogenic and commensal gut bacteria. Percentage of strains identified to have various putrefaction pathways in **(A)** pathogenic and **(B)** commensal gut bacteria. Pathway distribution in individual strains of the **(C)** pathogenic and **(D)** commensal gut bacteria.

The analysis of pathogenic and commensal gut bacteria revealed that, all the 21 strains affiliated as pathogens contain one or more putrefaction pathways (**Figure 4**). The three pathways occurring in relatively higher number of pathogens include putrescine, spermidine/spermine production and histidine degradation (predicted in ∼90%, ∼60% and ∼50%, respectively) (**Figure 4A**). In contrast, only the pathways for H_2_S and putrescine production were predicted in around 30% of the commensals (**Figure 4B**). Apart from the toxic effect on colonic cells, H_2_S has been proposed to aid bacteria in the formation of biofilm as well as benefit the host cell through mediating production of colonic mucus layer (Motta et al., 2015). Thus, the occurrence of H_2_S production pathway in commensal strains of *Bifidobacterium* and *Coprococcus* may reflect their own survival strategies and/or their beneficial effects on host cell. Most of the pathogenic putrescine producers (61%), belonging to the family Enterobacteriaceae, were found to have the pathway involving agmatinase (arginine → agmatine → putrescine) (Supplementary Figure S1). Earlier studies have also reported the role of this pathway in contamination of food by these groups of organisms (Wunderlichová et al., 2014). On the other hand, all the four commensal putrescine producers were predicted to utilize either ornithine decarboxylase (ODC) (arginine → ornithine → putrescine) or carbamoylputrescine hydrolase (arginine → agmatine →*N*-carbamoylputrescine → putrescine) pathway (Supplementary Figure S1). These observations suggest that, pathogen and commensal gut bacteria probably prefer different pathways for production of putrescine through arginine fermentation. Another interesting observation pertains to the organization of the genes corresponding to ODC pathway. Among the bacteria producing putrescine through the utilization of this pathway, only the pathogenic strains under *Fusobacterium* genus were observed to possess a single fused gene (encoding a single protein containing two functional domains, ‘Arginase’ and ‘OKR_DC_1’) (Supplementary Figure S2). In contrast, other organisms (pathogenic strains under *Clostridium* genus and commensal strains under *Faecalibacterium* genus) were identified to have these two domains as part of two different proteins encoded by separate genes (Supplementary Figure S2). Gene fusion has been reported earlier to confer a transcriptional advantage for synthesis of the corresponding proteins (Long, 2000). Thus, the functional potential of ODC pathway in *Fusobacterium* may be higher than others. Another pathway, prevalent in the pathogens, pertains to histidine degradation (leading to release of ammonia), which was predicted in 50% of the pathogenic strains (**Figures 4A,C**). The results suggest that, organisms under Enterobacteriaceae family (corresponding to the genera *Citrobacter, Enterobacter, Klebsiella, Salmonella*, and *Vibrio*) and the genus *Bacillus* probably utilize formiminoglutamase (EC 3.5.3.8) for catalyzing one of the reactions (*N*-formimino-L-glutamate →L-glutamate) of the pathway. The remaining pathogenic strains belonging to the genera *Fusobacterium* and *Bacteroides* were predicted to utilize an alternate enzyme, namely glutamate formyltransferase (EC 2.1.2.5), for catalyzing the same reaction. These observations indicate that, during evolution different bacterial groups may have acquired different genetic elements for fermenting same amino acid (like arginine and histidine). The remaining seven putrefaction pathways, namely glutamate degradation and production of THF, cadaverine, cresol, indole, phenol and H_2_S, were observed to be present in relatively lesser proportion (≤24%) of the pathogens (**Figures 4A,C**). Earlier studies have implicated the involvement of some of the pathogens like *Salmonella Typhimurium*, *Bacillus cereus*, *Escherichia coli* O157:H7 in contamination and fermentation of meat leading to release of harmful toxins, which has been reported to cause enteric inflammation (Lawrie and Ledward, 2006). Thus, the insights obtained from the current analysis suggest possible role of two of the putrefaction pathways, namely, histidine degradation and spermidine/spermine production (occurring in ∼50% and 60%, respectively, in the analyzed pathogenic strains), in the pathogenicity of gut bacteria.

### Enrichment of Putrefaction Pathways (Associated with Release of Harmful Compounds) in Gut Microbiome of Colorectal Cancer Patients

**FIGURE 5:**
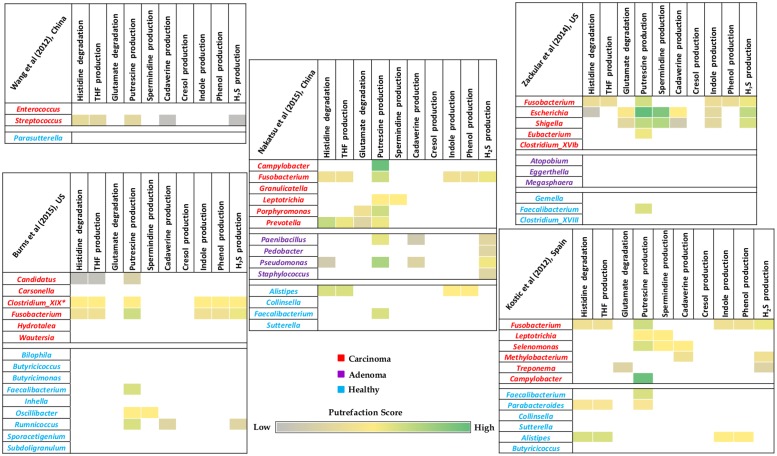
Putrefaction capabilities of differentially abundant genera in gut microbiome of healthy and colorectal cancer patients. Heatmaps representing distribution of selected putrefaction pathways (associated with production of harmful compounds) in the differentially abundant genera in one of the cohorts (carcinoma/adenoma/healthy) with respect to the other(s). Each cell represents the ‘Pfacs’ (Putrefaction score) of the corresponding pathway in that particular genus. ‘Pfacs’ indicates the relative putrefaction capabilities of any genus, evaluated based on the number of constituting putrefying strains and the database size of the respective genus.

Given the putrefying capabilities of gut associated pathogenic organisms (as discussed in section “Gut Bacteria and Putrefaction Pathways Associated with Release of Harmful Compounds”) and the potential role of some of the putrefaction products in disease etiology of CRC, a comparative analysis was performed on publicly available gut microbiome data of CRC patients and corresponding healthy controls. The differentially abundant bacterial genera in these cohorts were considered for capturing possible distinctness in the putrefaction capabilities of their gut microbiomes. A relative enrichment of putrefying bacteria was observed in carcinoma microbiome as compared to that in healthy samples (**Figure 5**). Out of the 10 analyzed putrefaction pathways, the differentially abundant genera in carcinoma and healthy cohorts were observed to represent nine and seven pathways, respectively. However, the percentage of occurrence of these pathways in the carcinoma cohort was relatively higher compared to that in the healthy cohort. For example, although putrescine production pathway was observed to have the highest occurrence in both the cohorts, it was found to be present in 58% (occurring in 11 out of the 19 differentially abundant genera) and 26% (occurring in 4 out of the 15 differentially abundant genera) in carcinoma and healthy cohorts, respectively. The next highest occurring pathway in carcinoma cohort was found to be histidine degradation (31.5%), occurrence of which was seen to be only 13.3% in healthy cohort. Considering the differentially abundant genera in carcinoma samples, the genus *Fusobacterium*, containing six of the analyzed putrefaction pathways (distributed among its strains), was observed to be differentially abundant in four out the five datasets. The putrefaction capability of *Fusobacterium* has also been implicated in promoting periodontitis in oral cavity (Flynn et al., 2016). Apart from *Fusobacterium*, some of the other genera (such as *Streptococcus, Candidatus, Escherichia, Shigella*, *Prevotella, Selenomonas*, and *Clostridium_XIX*), predicted to possess at least three putrefaction pathways, were observed to be significantly higher in carcinoma microbiome. It may be noted that, the completely sequenced strains belonging to the ‘*Clostridium_XIX*’ group corresponded to those under *Fusobacterium nucleatum* species (Xing et al., 2011). Consequently, in this analysis, ‘*Clostridium_XIX*’ has been considered to represent a subset of *Fusobacterium* genus. In contrast to the carcinoma cohort, only three differentially abundant genera in healthy cohort, namely, *Alistipes, Parabacteroides*, and *Ruminococcus*, were predicted to have at least three of the analyzed putrefaction pathways. Among these, *Alistipes* was observed to have highest number of pathways which included histidine degradation and productions of THF, indole, and phenol. This genus, although generally considered as a commensal, has been implicated in inflammation and bacteremia in patients of gastrointestinal disorders (Fenner et al., 2007; Jiang et al., 2015). Thus, the identified putrefaction pathways in *Alistipes* may contribute in deleterious consequences under dysbiotic conditions. Apart from putrescine production pathway which was found to be common in *Parabacteroides* and *Ruminococcus*, the former was predicted to harbor histidine degradation as well as THF production pathways and the latter was seen to have cadaverine and H_2_S production pathways. Organisms belonging to *Parabacteroides* have been reported to carry resistance genes against antimicrobial drugs and consequently behave as opportunistic pathogens (Boente et al., 2010). Thus, it is possible that the identified putrefaction pathways in *Parabacteroides* may function only under certain conditions, when the bacteria exhibit pathogenicity. The other two genera (in addition to the above mentioned three genera) which were found to be differentially abundant in healthy cohort, namely, *Oscillibacter* (containing two pathways) and *Faecalibacterium* (containing one pathway), were predicted to have putrescine production pathway in common and spermidine/spermine production as an additional pathway in the former. Earlier studies have indicated importance of metabolites like putrescine and H_2_S in functions like growth and biofilm formation in bacteria (Wortham et al., 2007; Shatalin et al., 2011). Thus, it is likely that the putrefaction pathways producing these metabolites by the above mentioned four genera (*Faecalibacterium, Oscillibacter*, *Ruminococcus*, and *Parabacteroides*) may be utilized by the bacteria for their own benefit, rather than exerting harmful effects in the healthy cohort. Additionally, three of the above mentioned genera from healthy cohort (*Ruminococcus*, *Faecalibacterium*, and *Oscillibacter*) have been reported to produce butyrate, which is known to participate in multiple functions (including anti-inflammatory role) that are beneficial to gut health (Anand et al., 2016; Rivière et al., 2016). Overall, the observation of relatively higher percentage of histidine degradation and putrescine production pathways in carcinoma cohort, combined with the results of the analysis on gut pathogens (as discussed in the previous section) indicate possible role of these pathways in disease pathogenesis of CRC.

### Hypoxic Tumor Microenvironment and Putrefying Bacteria

**FIGURE 6:**
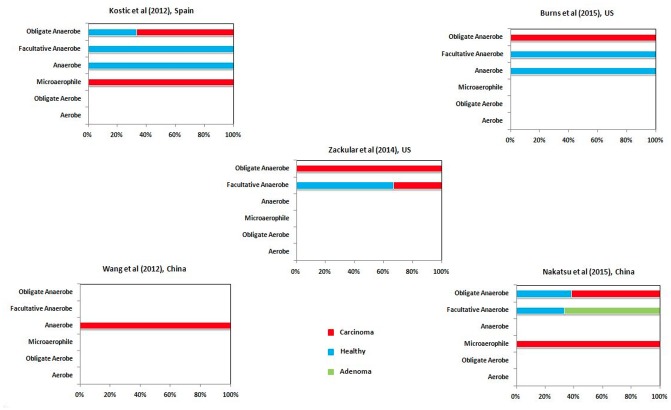
Correlation between putrefaction and oxygen requirement. Percentage of differential putrefying genera in the gut microbiome of healthy, adenoma and carcinoma datasets in different categories (based on oxygen requirement).

The results of the microbiome analysis indicated a relative enrichment of putrefying bacteria in gut microbiome of CRC cohort at later stage of the disease as compared to that in the early adenoma stage (**Figure 5**). Advanced stage of the disease has been shown earlier to be correlated with hypoxic (oxygen deficit) microenvironment and consequent growth of anaerobic pathogenic bacteria (Scanlon and Glazer, 2015). The current results suggest that, the anaerobic bacterial genera (especially obligate anaerobes and microaerophiles) which are differentially abundant in carcinoma microbiome are mostly putrefactors, as opposed to that of healthy population in all datasets examined in this study (**Figure 6**). Interestingly, while the differentially abundant anaerobic genera corresponding to one of the adenoma cohort were predicted to have only histidine degradation and production of putrescine, cadaverine, and H_2_S, the other dataset lacked any putrefaction pathway. The decreased number of putrefaction pathways in adenoma (as compared to the healthy samples), as observed from the analysis based on the limited number of available datasets, would require further validation on a larger cohort. Such studies are likely to provide better insights on the cause-consequence relationship between hypoxic tumor micro-environment and putrefaction by anaerobic bacteria.

## Discussion

The associations between gut bacteria and human health are being increasingly studied for understanding various diseases/disorders. In this study, gut microbiome’s potential in protein fermentation process (i.e., putrefaction) which leads to release of harmful metabolites has been computationally analyzed. The results of our analyses suggest a relatively higher putrefaction abilities of the phyla that are commonly found in the gut (such as Firmicutes, Bacteroidetes, Actinobacteria, Proteobacteria, and Fusobacteria), indicating significance of bacterial putrefaction in gut environment. Further, an interesting observation corresponds to the comparative ‘Pfacs’ (Putrefaction score) of Bacteroidetes and Firmicutes. The scores for most of the individual pathways (degradation of histidine, production of THF, putrescine, indole, and phenol) were observed to be higher in Bacteroidetes than Firmicutes. Earlier experimental studies have reported increase in Bacteroidetes to Firmicutes ratio in dysbiotic gut (Wills et al., 2014; Kabeerdoss et al., 2015). Thus, the current observations suggest possible contribution of putrefaction capabilities of Bacteroidetes in disruption of gut homeostasis. In addition, the results indicate probable role of selected putrefaction pathways (ammonia releasing histidine degradation pathway and the pathway for spermidine/spermine production) in pathogenicity of the resident gut bacteria. In addition, the analysis of gut microbiome of CRC patients indicates probable involvement of the selected putrefaction pathways in the disease etiology of CRC. It may further be noted that, the observed relative enrichment of putrefaction pathways in carcinoma cohort could be associated to intestinal inflammation that may promote the growth of putrefying bacteria. However, it is difficult to infer from the present study the cause/consequence relationship between CRC and bacterial putrefaction. Further experimental studies would be required to validate the same. Thus, such phenomenon is possibly a consequence of the tumor micro-environment rather than a cause.

It is noteworthy that products/by-products generated by bacterial putrefaction are harmless to human body when present within a certain level. Under such conditions, ammonia produced during putrefaction enters urea or ornithine cycle to get converted to urea, which gets excreted from the system (Katayama, 2016). Also, certain amount of indole and putrescine are sometimes beneficial to gut health. Indole has been reported to contribute in defense against intestinal worms (Anyanful et al., 2005). Putrescine has been suggested to exert anti-inflammatory activities and help in proper functioning of intestinal mucosal barrier function (Deloyer et al., 2005). However, elevated levels of the putrefaction products have been implicated in disruption of gut homeostasis and also in colorectal tumor progression, especially in individuals with compromised immune system and prior exposure to gut infection/diseases (Hughes et al., 2000; Kim et al., 2013). This observation, in combination with the insights obtained from the current study (indicating probability of production of the aforementioned compounds in higher amounts in CRC patients) suggests the possible involvement of putrefaction pathways in the disease pathogenesis.

One of the key results obtained from the current study pertains to the genus *Fusobacterium* which was observed to be not only significantly abundant in majority of the carcinoma datasets, but was also observed to have higher number of putrefaction pathways which lead to release of harmful metabolites. *Fusobacterium* has also been identified as a risk factor for colorectal carcinoma progression (Li et al., 2016; Yang et al., 2016). An adhesion factor FadA from *Fusobacterium nucleatum* has been suggested to induce colorectal carcinogenesis in animal models (Allen-Vercoe and Jobin, 2014). This bacterium has also been shown to exhibit immunosuppressive effects on host T-cell responses (Nosho et al., 2016). Earlier studies have also suggested role of this organism inhabiting the oral cavity in aggravation of periodontal infections through protein fermentation (Flynn et al., 2016). Interestingly, unlike commensal gut bacteria, *Fusobacterium* has been shown to utilize an ammonia releasing pathway for production of butyrate which plays multiple beneficial roles in gut (Anand et al., 2016). Thus, in spite of being a butyrate producer, this bacterial group has been speculated to harm gut homeostasis by releasing ammonia. Therefore, similar to the above mentioned aspects of *Fusobacterium* genus, the results of the present study indicate probable role of putrefaction capability of this bacterial group in pathogenesis of CRC. The insights from the current study shed light on importance of bacterial putrefaction pathways in CRC augmentation and progression. Further experimental validations of the findings are expected to help in development of microbiome based diagnostic/therapeutic strategies for effective control of CRC.

## Author Contributions

HK, CD, and SM conceptualized the work and designed the experiments. HK and CD performed the experiments. HK, CD, and SM analyzed the data and drafted the manuscript. All authors reviewed the results and approved the final manuscript.

## Conflict of Interest Statement

The authors declare that the research was conducted in the absence of any commercial or financial relationships that could be construed as a potential conflict of interest.

## Footnotes

1 ftp://ftp.ncbi.nlm.nih.gov/genomes/genbank/bacteria

2 http://www.uniprot.org/

3 http://www.uniprot.org/
