# Supplementary material for: In Silico Analysis of Putrefaction Pathways in Bacteria and Its Implication in Colorectal Cancer
Source: Front Microbiol. 2017 Nov 7;8:2166. doi: 10.3389/fmicb.2017.02166 (PMC5682003; doi:10.3389/fmicb.2017.02166)
Supplement: Supplementary file 1 [file Table_1.PDF]

Table S1: Details on the organisms where the putrefaction pathways have been identified experimentally.

Putrescine_1, Putrescine_2 and Putrescine_3 represent the pathways involving ornithine decarboxylase (ODC), agmatinase and carbamoylputrescine hydrolase respectively.

Putrefaction Pathways		Reference organism	Reference
**Histidine degradation** (Histidine → glutamate)		*Pseudomonas fluorescens* SBW25, *Fusobacterium nucleatum* ATCC 25586	Zhang and Rainey, 2007; Kastenmüller et al., 2009
**THF production** (Histidine → Tetrahydrofolate)		*Fusobacterium nucleatum* ATCC 25586	Kastenmüller et al., 2009
**Glutamate degradation** (glutamate → acetate + pyruvate)		*Fusobacterium varium* ATCC 27725	Ramezani et al., 2011
**Putrescine production** (arginine → putrescine)	Putrescine_1	*Lactobacillus sp*. 30a, *Oenococcus oeni* and *Lacobacillus brevis* IOEB 9906	Coton et al., 2010; Romano et al., 2014
	Putrescine_2	*Escherichia coli*	Satishchandran and Boyle, 1986
	Putrescine_3	*Pseudomonas aeruginosa* PAO1	Nakada et al., 2001
**Spermidine/Spermine production** (methionine → spermidine/spermine)		*Escherichia coli*	Shah et al., 2008
**Cresol production** (tyrosine → cresol)		*Clostridium difficile*	Dawson et al., 2011

References

Coton, E., Mulder, N., Coton, M., Pochet, S., Trip, H., and Lolkema, J. S. (2010). Origin of the putrescine-producing ability of the coagulase-negative bacterium Staphylococcus epidermidis 2015B. *Appl. Environ. Microbiol.* 76, 5570–5576. doi:10.1128/AEM.00441-10.

Dawson, L. F., Donahue, E. H., Cartman, S. T., Barton, R. H., Bundy, J., McNerney, R., et al. (2011). The analysis of para-cresol production and tolerance in Clostridium difficile 027 and 012 strains. *BMC Microbiol.* 11, 86. doi:10.1186/1471-2180-11-86.

Kastenmüller, G., Schenk, M. E., Gasteiger, J., and Mewes, H.-W. (2009). Uncovering metabolic pathways relevant to phenotypic traits of microbial genomes. *Genome Biol.* 10, R28. doi:10.1186/gb-2009-10-3-r28.

Nakada, Y., Jiang, Y., Nishijyo, T., Itoh, Y., and Lu, C. D. (2001). Molecular characterization and regulation of the aguBA operon, responsible for agmatine utilization in Pseudomonas aeruginosa PAO1. *J. Bacteriol.* 183, 6517–6524. doi:10.1128/JB.183.22.6517-6524.2001.

Ramezani, M., Resmer, K. L., and White, R. L. (2011). Glutamate racemization and catabolism in Fusobacterium varium. *FEBS J.* 278, 2540–2551.

doi:10.1111/j.1742-4658.2011.08179.x.
Romano, A., Ladero, V., Alvarez, M. A., and Lucas, P. M. (2014). Putrescine production via the ornithine decarboxylation pathway improves the acid stress survival of Lactobacillus brevis and is part of a horizontally transferred acid resistance locus. *Int. J. Food Microbiol.* 175, 14–19. doi:10.1016/j.ijfoodmicro.2014.01.009.
Satishchandran, C., and Boyle, S. M. (1986). Purification and properties of agmatine ureohydrolyase, a putrescine biosynthetic enzyme in Escherichia coli. *J. Bacteriol.* 165, 843–848.
Shah, P., and Swiatlo, E. (2008). A multifaceted role for polyamines in bacterial pathogens. Mol. Microbiol. 68, 4–16. doi:10.1111/j.1365-2958.2008.06126.x.
Zhang, X.-X., and Rainey, P. B. (2007). Genetic Analysis of the Histidine Utilization (hut) Genes in Pseudomonas fluorescens SBW25. *Genetics* 176, 2165–2176. doi:10.1534/genetics.107.075713.
