# Supplementary material for: In Silico Analysis of Putrefaction Pathways in Bacteria and Its Implication in Colorectal Cancer
Source: Front Microbiol. 2017 Nov 7;8:2166. doi: 10.3389/fmicb.2017.02166 (PMC5682003; doi:10.3389/fmicb.2017.02166)
Supplement: Supplementary file 2 [file Table_2.PDF]

Table S2: Details on the enzymes involved in each of the ten putrefaction pathways.

Putrescine_1, Putrescine_2 and Putrescine_3 represent the pathways involving ornithine decarboxylase (ODC) (EC: 4.1.1.17), agmatinase (EC: 3.5.3.11) and carbamoylputrescine hydrolase (EC: 3.5.1.53) respectively. H_2S_1, H_2S_2, H_2S_3, H_2S_4 and H_2S_5 represent the pathways involving cystathionine gamma lyase (EC: 4.4.1.1), 3-mercaptopyruvate sulfurtransferase (EC: 2.8.1.2), cystathionine beta-synthase (EC: 4.2.1.22), L-cysteine desulfhydrase (EC: 4.4.1.28) and D-cysteine desulfhydrase (EC: 4.4.1.15) respectively.

Putrefaction pathways		EC Number	Pfam ID	Pfam Domain
**Histidine degradation (histidine → glutamate)**		4.3.1.3	PF00221	Lyase_aromatic
		4.2.1.49	PF01175	Urocanase
		3.5.2.7	PF01979	Amidohydro_1
		2.1.2.5	PF07837	FTCD_N
			PF02971	FTCD
		3.5.3.8	PF00491	Arginase
**THF production (histidine → tetrahydrofolate)**		4.3.1.3	PF00221	Lyase_aromatic
		4.2.1.49	PF01175	Urocanase
		3.5.2.7	PF01979	Amidohydro_1
		2.1.2.5	PF07837	FTCD_N
			PF02971	FTCD
		4.3.1.4	PF04961	FTCD_C
**Glutamate degradation (glutamate → acetate + pyruvate)**		5.4.99.1	PF06368	Met_asp_Mut_E
		4.3.1.2	PF07476	MAAL_C
			PF05034	MAAL_N
		4.2.1.34	PF00330	Aconitase
		4.1.3.22	PF13714	PEP_mutase
**Putrescine production (arginine → putrescine)**	**Putrescine_1**	3.5.3.1	PF00491	Arginase
		4.1.1.17	PF01276	OKR_DC_1
	**Putrescine_2**	4.1.1.19	PF02784	Orn_Arg_deC_N
			PF00278	Orn_DAP_Arg_deC
		3.5.3.11	PF00491	Arginase
	**Putrescine_3**	4.1.1.19	PF02784	Orn_Arg_deC_N
			PF00278	Orn_DAP_Arg_deC
		3.5.3.12	PF04371	PAD_porph
**Spermidine/Spermine production (methionine → spermidine/spermine)**		2.5.1.6	PF00438	S_AdoMet_synt_N
			PF02772	S_AdoMet_synt_M
			PF02773	S_AdoMet_synt_C
		4.1.1.50	PF02675	AdoMet_dc
		2.5.1.16	PF01564	Spermine_synth
**Cadaverine production (lysine → cadaverine)**		4.1.1.18	PF03709	OKR_DC_1_N
			PF01276	OKR_DC_1
			PF03711	OKR_DC_1_C
**Cresol production (tyrosine → cresol)**		2.6.1.5	PF00155	Aminotran_1_2
		2.6.1.1		
		2.6.1.9		
		2.6.1.58		
		2.6.1.57		
		4.1.1.80	PF02775	TPP_enzyme_C
			PF02776	TPP_enzyme_N
			PF00205	TPP_enzyme_M
		1.2.1.29	PF00171	Aldedh
		4.1.1.83	PF04055	Radical_SAM
			PF01228	Gly_radical
			PF02901	PFL-like
**Phenol production (tyrosine → phenol)**		4.1.99.2	PF01212	Beta_elim_lyase

Putrefaction pathways		EC Number	Pfam ID	Pfam Domain
**Indole production (tryptophan → indole)**		4.1.99.1	PF01212	Beta_elim_lyase
**H_2S production (cysteine → H_2S)**	**H_2S_1**	4.4.1.1	PF01053	Cys_Met_Meta_PP
	**H_2S_2**	2.8.1.2	PF00581	Rhodanese
	**H_2S_3**	4.2.1.22	PF00291	PALP
			PF00571	CBS
	**H_2S_4**	4.4.1.28	PF03313	SDH_alpha
	**H_2S_5**	4.4.1.15	PF00291	PALP
