# Supplementary material for: In Silico Analysis of Putrefaction Pathways in Bacteria and Its Implication in Colorectal Cancer
Source: Front Microbiol. 2017 Nov 7;8:2166. doi: 10.3389/fmicb.2017.02166 (PMC5682003; doi:10.3389/fmicb.2017.02166)
Supplement: Supplementary file 3 [file Table_3.PDF]

Table S3: List of pathogenic and commensal bacteria that have been experimentally reported to be associated with human gut.

Gut Pathogen	Reference
Bacillus_cereus_AH820_uid58751	Kotiranta et al., 2000
Bacillus_cereus_G9842_uid58759	Kotiranta et al., 2000
Bacteroides_thetaiotaomicron_VPI_5482_uid62913	Chen et al., 2017
Bacteroides_vulgatus_ATCC_8482_uid58253	Chen et al., 2017
Campylobacter_concisus_13826_uid58667	Chen et al., 2017
Citrobacter_koseri_ATCC_BAA_895_uid58143	Putze et al., 2009
Clostridium_difficile_BI1_uid158363	He et al., 2010
Clostridium_difficile_CD196_uid41017	Stabler et al., 2009
Enterobacter_cloacae_NCTC_9394_uid197202	Veleba et al., 2013
Escherichia_coli_O157_H7_EC4115_uid59091	Eppinger et al., 2011
Escherichia_coli_042_uid161985	Chaudhuri et al., 2010
Fusobacterium_3_1_36A2_uid55995	Chen et al., 2017
Fusobacterium_4_8_uid205051	Chen et al., 2017
Fusobacterium_nucleatum_ATCC_25586_uid57885	Chen et al., 2017
Klebsiella_pneumoniae_NTUH_K2044_uid59073	Wu et al., 2009
Salmonella_enterica_serovar_Paratyphi_A_AKU_12601_uid59269	Chen et al., 2017
Salmonella_enterica_serovar_Paratyphi_A_ATCC_9150_uid58201	Chen et al., 2017
Salmonella_enterica_serovar_Typhimurium_T000240_uid84397	Izumiya et al., 2011
Shigella_dysenteriae_Sd197_uid58213	Yang et al., 2005
Shigella_flexneri_2a_2457T_uid57991	Chen et al., 2017
Vibrio_cholerae_O1_2010EL_1786_uid78933	Reimer et al., 2011
Vibrio_cholerae_O1_biovar_El_Tor_N16961_uid57623	Heidelberg et al., 2000

Gut Commensal	Reference
Bifidobacterium_animalis_lactis_V9_uid158865	Chen et al., 2017
Bifidobacterium_bifidum_BGN4_uid167988	
Bifidobacterium_bifidum_PRL2010_uid59883	
Bifidobacterium_longum_NCC2705_uid57939	
Campylobacter_hominis_ATCC_BAA_381_uid58981	
Coprococcus_ART55_1_uid197176	
Coprococcus_catus_GD_7_uid197174	
Faecalibacterium_prausnitzii_L2_6_uid197183	
Lactobacillus_rhamnosus_GG_uid161983	
Lactobacillus_rhamnosus_GG_uid59313	
Roseburia_hominis_A2_183_uid73419	
Roseburia_intestinalis_uid197164	

References

Chaudhuri, R. R., Sebaihia, M., Hobman, J. L., Webber, M. A., Leyton, D. L., Goldberg, M. D., et al. (2010). Complete genome sequence and comparative metabolic profiling of the prototypical enteroaggregative Escherichia coli strain 042. *PloS One* 5, e8801. doi:10.1371/journal.pone.0008801.

Chen, I.-M. A., Markowitz, V. M., Chu, K., Palaniappan, K., Szeto, E., Pillay, M., et al. (2017). IMG/M: integrated genome and metagenome comparative data analysis system. *Nucleic Acids Res.* 45, D507–D516. doi:10.1093/nar/gkw929.

Eppinger, M., Mammel, M. K., Leclerc, J. E., Ravel, J., and Cebula, T. A. (2011). Genomic anatomy of Escherichia coli O157:H7 outbreaks. *Proc. Natl. Acad. Sci. U. S. A.* 108, 20142–20147. doi:10.1073/pnas.1107176108.

He, M., Sebaihia, M., Lawley, T. D., Stabler, R. A., Dawson, L. F., Martin, M. J., et al. (2010). Evolutionary dynamics of Clostridium difficile over short and long time scales. *Proc. Natl. Acad. Sci. U. S. A.* 107, 7527–7532. doi:10.1073/pnas.0914322107.

Heidelberg, J. F., Eisen, J. A., Nelson, W. C., Clayton, R. A., Gwinn, M. L., Dodson, R. J., et al. (2000). DNA sequence of both chromosomes of the cholera pathogen Vibrio cholerae. *Nature* 406, 477–483. doi:10.1038/35020000.

Izumiya, H., Sekizuka, T., Nakaya, H., Taguchi, M., Oguchi, A., Ichikawa, N., et al. (2011). Whole-Genome Analysis of Salmonella enterica Serovar Typhimurium T000240 Reveals the Acquisition of a Genomic Island Involved in Multidrug Resistance via IS1 Derivatives on the Chromosome. *Antimicrob. Agents Chemother.* 55, 623–630. doi:10.1128/AAC.01215-10.

Kotiranta, A., Lounatmaa, K., and Haapasalo, M. (2000). Epidemiology and pathogenesis of Bacillus cereus infections. *Microbes Infect.* 2, 189–198.

Putze, J., Hennequin, C., Nougayrède, J.-P., Zhang, W., Homburg, S., Karch, H., et al. (2009). Genetic Structure and Distribution of the Colibactin Genomic Island among Members of the Family Enterobacteriaceae. *Infect. Immun.* 77, 4696–4703. doi:10.1128/IAI.00522-09.

Reimer, A. R., Van Domselaar, G., Stroika, S., Walker, M., Kent, H., Tarr, C., et al. (2011). Comparative genomics of Vibrio cholerae from Haiti, Asia, and Africa. *Emerg. Infect. Dis.* 17, 2113–2121. doi:10.3201/eid1711.110794.

Stabler, R. A., He, M., Dawson, L., Martin, M., Valiente, E., Corton, C., et al. (2009). Comparative genome and phenotypic analysis of Clostridium difficile 027 strains provides insight into the evolution of a hypervirulent bacterium. *Genome Biol.* 10, R102. doi:10.1186/gb-2009-10-9-r102.

Veleba, M., De Majumdar, S., Hornsey, M., Woodford, N., and Schneiders, T. (2013). Genetic characterization of tigecycline resistance in clinical isolates of Enterobacter cloacae and Enterobacter aerogenes. *J. Antimicrob. Chemother.* 68, 1011–1018. doi:10.1093/jac/dks530.

Wu, K.-M., Li, L.-H., Yan, J.-J., Tsao, N., Liao, T.-L., Tsai, H.-C., et al. (2009). Genome sequencing and comparative analysis of Klebsiella pneumoniae NTUH-K2044, a strain causing liver abscess and meningitis. *J. Bacteriol.* 191, 4492–4501. doi:10.1128/JB.00315-09.

Yang, F., Yang, J., Zhang, X., Chen, L., Jiang, Y., Yan, Y., et al. (2005). Genome dynamics and diversity of Shigella species, the etiologic agents of bacillary dysentery. *Nucleic Acids Res.* 33, 6445–6458. doi:10.1093/nar/gki954.
