# Supplementary material for: In Silico Analysis of Putrefaction Pathways in Bacteria and Its Implication in Colorectal Cancer
Source: Front Microbiol. 2017 Nov 7;8:2166. doi: 10.3389/fmicb.2017.02166 (PMC5682003; doi:10.3389/fmicb.2017.02166)
Supplement: Supplementary file 4 [file Table_4.PDF]

**Table S4: Details on the 16S rRNA datasets analyzed in the current study**

| Serial No. | No. of samples | Sample type | Geography | Reference | SRA ID/ Link to the SRA files |
|---|---|---|---|---|---|
| 1. | 90 (30 healthy, 30 adenoma, 30 carcinoma) | Stool | US | Zackular et al., 2014 | http://www.mothur.org/MicrobiomeBiomarkerCRC |
| 2. | 88 (44 normal, 44 tumor ) | Tissue | US | Burns et al., 2015 | PRJNA284355 |
| 3. | 102 (46 healthy, 56 carcinoma) | Stool | China | Wang et al., 2012 | SRP005150 |
| 4. | 160 (61 healthy, 47 adenoma , 52 tumor) | Tissue | China | Nakatsu et al., 2015 | PRJNA280026 |
| 5. | 190 (95 normal, 95 tumor) | Tissue | Spain | Kostic et al., 2012 | SRP000383 |

**References**

Burns, M. B., Lynch, J., Starr, T. K., Knights, D., and Blekhman, R. (2015). Virulence genes are a signature of the microbiome in the colorectal tumor microenvironment. *Genome Med.* 7, 55. doi:10.1186/s13073-015-0177-8.

Kostic, A. D., Gevers, D., Pedamallu, C. S., Michaud, M., Duke, F., Earl, A. M., et al. (2012). Genomic analysis identifies association of Fusobacterium with colorectal carcinoma. *Genome Res.* 22, 292–298. doi:10.1101/gr.126573.111.

Nakatsu, G., Li, X., Zhou, H., Sheng, J., Wong, S. H., Wu, W. K. K., et al. (2015). Gut mucosal microbiome across stages of colorectal carcinogenesis. *Nat. Commun.* 6, 8727. doi:10.1038/ncomms9727.

Wang, T., Cai, G., Qiu, Y., Fei, N., Zhang, M., Pang, X., et al. (2012). Structural segregation of gut microbiota between colorectal cancer patients and healthy volunteers. *ISME J.* 6, 320–329. doi:10.1038/ismej.2011.109.

Zackular, J. P., Rogers, M. A. M., Ruffin, M. T., and Schloss, P. D. (2014). The human gut microbiome as a screening tool for colorectal cancer. *Cancer Prev. Res. Phila. Pa* 7, 1112–1121. doi:10.1158/1940-6207.CAPR-14-0129.
