# Supplementary figures and images for: In Silico Analysis of Putrefaction Pathways in Bacteria and Its Implication in Colorectal Cancer
Source: Front Microbiol. 2017 Nov 7;8:2166. doi: 10.3389/fmicb.2017.02166 (PMC5682003; doi:10.3389/fmicb.2017.02166)

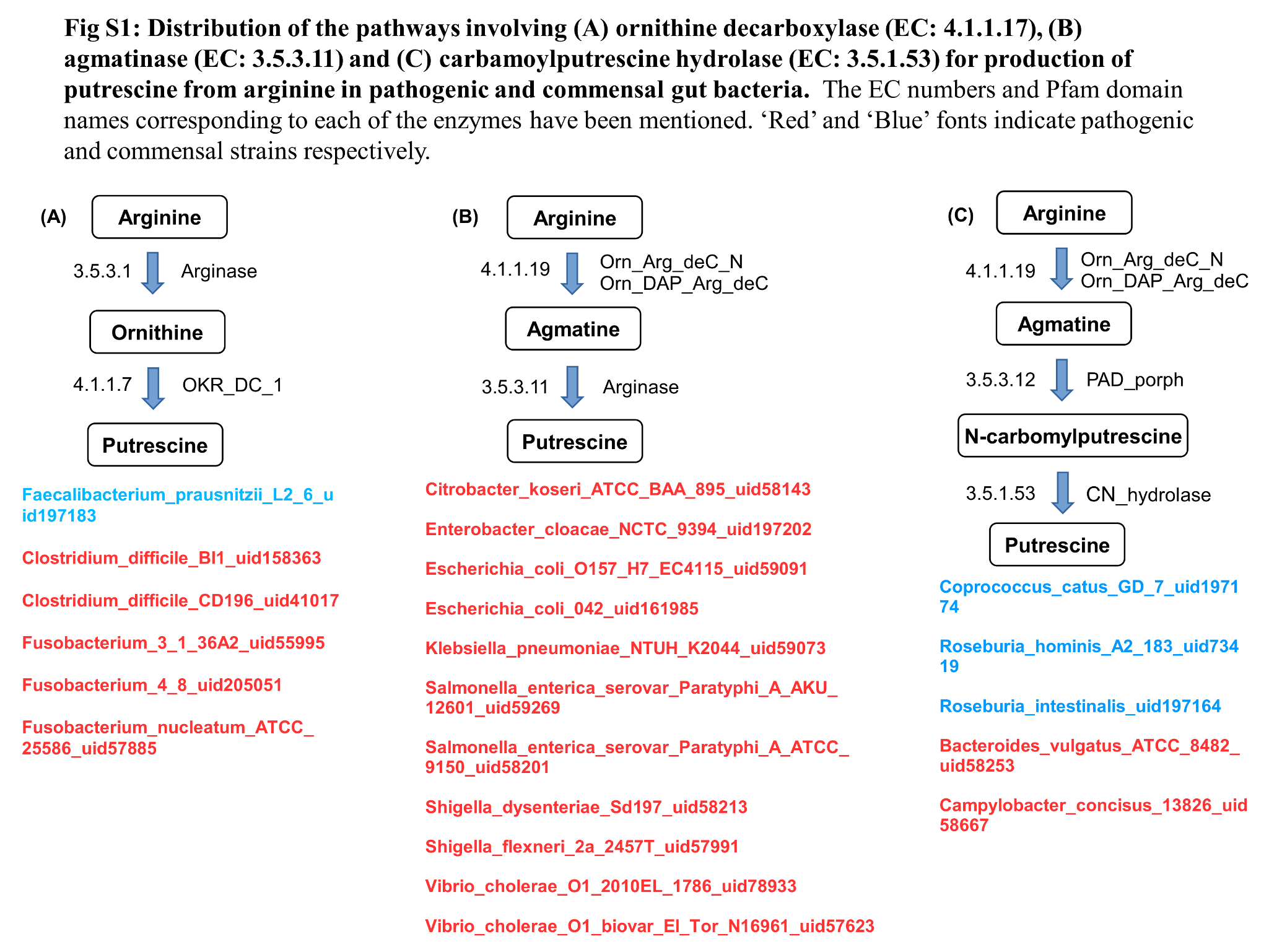

Supplement: Supplementary file 6 [file Image_1.TIFF]

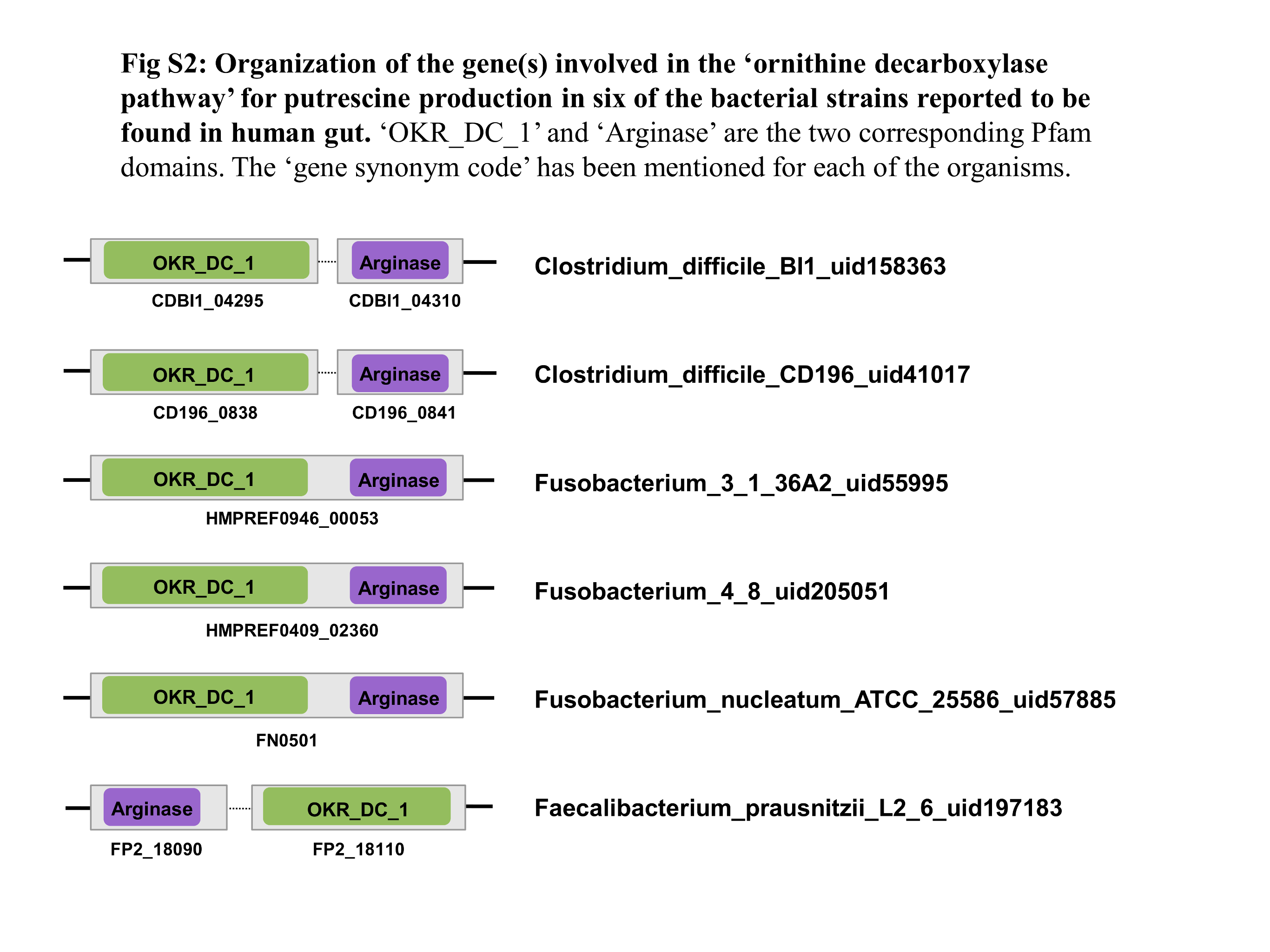

Supplement: Supplementary file 7 [file Image_2.TIFF]
